# Impact of hormones on lipedema development: a systematic literature review

**DOI:** 10.1007/s00404-026-08318-1

**Published:** 2026-01-23

**Authors:** Julia Elisabeth Lüchinger, Elena Pavicic, Cynthia Laura Giachino, Petra Stute

**Affiliations:** 1 Faculty of Medicine, University of Bern, Bern, Switzerland; 2 Department of Obstetrics and Gynecology, University Hospital of Bern, Bern, Switzerland; 3 Graduate School for Health Sciences, University of Bern, Bern, Switzerland

**Keywords:** Lipedema, Hormones, Subcutaneous fat tissue, Estrogen

## Abstract

**Purpose:**

Lipedema is a chronic disorder that affects the subcutaneous adipose tissue of the lower and upper limbs and results in painful fat accumulations. During the reproductive life span, about 11% of women are affected; however, there are a high number of suspected undiagnosed and thus untreated cases.

**Methods:**

The aim of this systematic review was to evaluate the association between hormones and the pathophysiological mechanisms of lipedema development. Inclusion criteria were: lipedema, lipoedema, estrogen, estrogen antagonists, female sex hormones, hormones, insulin, puberty, pregnancy, menopause, subcutaneous fat tissue, and subcutaneous adipose connective tissue.

**Results:**

The literature search yielded 121 hits; after deduplication, 64 records were screened. After abstract and full-text screening 15 publications were suitable for being included in the systematic review. Overall, four different pathophysiological hypotheses were postulated: (1) general hormonal imbalance, (2) changes in growth hormone balance, (3) metabolic imbalance such as changes in adipose stem cells in relation to adipokines or leptin in association with the transcription factor PPARγ, and (4) changes in estrogen metabolism as well as alterations in the function of estrogen receptors.

**Conclusion:**

Lipedema appears to be a multifactorial condition primarily driven by hormonal dysregulation—especially involving estrogen—alongside metabolic and possible genetic components. The findings support the reclassification of lipedema as a hormonally influenced disorder distinct from obesity, emphasizing the need for further research into diagnostic biomarkers, targeted therapies, and the role of genetic susceptibility.

**Supplementary Information:**

The online version contains supplementary material available at 10.1007/s00404-026-08318-1.

## Introduction

Lipedema is a chronic condition affecting the subcutaneous fatty tissue of the predominantly female population, characterized by disproportionate and painful accumulations of fatty tissue in the lower and upper extremities [1]. According to Bauer et al. [2], 11% of all women, including post-pubertal girls, are affected by lipedema, although a significant number of cases remain unreported. The true prevalence may be considerably higher due to widespread underdiagnosis or misdiagnosis, driven by the fact that universally accepted diagnostic criteria are lacking [3]. As lipedema is frequently misdiagnosed with obesity or lymphedema, accurate differentiation is essential to ensure appropriate treatment [4, 5]. Furthermore, lipedema manifests in diverse morphological forms, impacting both physical and psychological well-being; common co-morbidities include anxiety, depression, and pain accompanying the primary physical discomfort, such as easy bruising, tenderness, and swelling [4, 6]. Despite the personal and public health implications, lipedema remains poorly understood [7]. Given the striking female predominance and frequent onset around hormonal transition phases—such as puberty, pregnancy, or menopause—researchers increasingly suspect that endocrine mechanisms may underlie or exacerbate lipedema [8]. Previous studies have shown associations between lipedema and hormonal change [8–10]. Particular emphasis has been placed on concepts involving female sex hormones, especially estrogen and progesterone, and their interactions with their respective receptors [3, 4, 6, 11–15]. Some studies also explore alternative pathophysiological hypotheses, such as alterations in growth hormone balance or metabolic hormones in connection with leptin and PPARγ [2, 7, 16, 17]. Clarifying these hormonal influences is essential for several clinical reasons: First, it could help to explain why conventional weight-management approaches often fail in lipedema patients, establishing lipedema as a distinct disorder rather than a variant of general obesity [3]. Second, understanding the hormonal pathophysiology may enable the development of hormone-based biomarkers for improved diagnostic accuracy, addressing the current challenge of clinical misdiagnosis [8]. Deeper understanding of pathophysiological connections—especially in estrogen receptor and metabolic pathways—could identify new therapeutic targets, potentially leading to pharmacological interventions beyond current symptomatic management strategies, such as compression therapy and liposuction [4]. These findings highlight the ongoing need to enhance awareness and deepen scientific understanding of lipedema, covering the full spectrum from its underlying causes and mechanisms to effective diagnosis and treatment strategies. To contribute to this long-term endeavor and provide a foundation for translating hormonal insights into clinical applications, this systematic literature review was conducted to consolidate existing literature on the pathophysiology of lipedema, with a focus on its hormonal associations and their potential clinical implications.

## Methods

### Information sources and search strategy

Extensive literature searches were created and executed by a medical information specialist for the following data sources to identify all potentially relevant papers on the investigated question: 1) MEDLINE (Ovid), 2) Embase (Ovid), 3) Cochrane Library (Wiley), 4) Science Citation Index Expanded; SCIE (Clarivate Analytics), and 5) Emerging Sources Citation Index; ESCI (Web of Science). An initial search strategy was conducted in MEDLINE by a medical information specialist to examine the initial criteria for conformity. After refinement and final adjustments, the complex literature search was implemented by combining terms from the controlled vocabulary (e.g., MeSH and EMTREE) and free text terms for the following concepts: Hormones and Lipedema. The search was conducted on April 5, 2023. No restrictions were placed on language or publication date. The search strategies were translated accordingly for each information source. Complete search strategies for all databases are documented in Supplementary File 1.

### Eligibility criteria

Articles were included if they mentioned the term lipedema or lipoedema. While the literature employs both "lipedema" and "lipoedema" interchangeably with identical meaning, “lipedema” is used consistently throughout this manuscript. Where cited articles use “lipoedema”, the spelling has been adapted to match the terminology in this review. Additionally, all articles referring to hormones, especially female sex hormones and phases of hormonal alterations, have been included (estrogen, estrogen antagonists, insulin, puberty, pregnancy, and menopause). The subcutaneous fat tissue or connective tissue represents a further inclusion criterion. As it is important to differentiate between various manifestations of oedema, articles mentioning a non-lipedema associated form of oedema were excluded (heart insufficiency, cardiac decompensation, hepatorenal syndrome, adrenal insufficiency, and ACTH).

### Study selection and data collection process

All included records were imported into Endnote and deduplicated using the automated deduplication software Deduklick. Title and abstract screening were carried out by two co-authors using Covidence. The full-text screening of each article was carried out by the same two co-authors. Any discrepancy was discussed by the co-authors, and for clarification, a third rater was involved. In a further step, all relevant data were extracted. The results were visualized graphically (PRISMA diagram, Supplementary File 2).

### Data outcomes

The systematic literature research initially yielded 121 hits. After deduplication a total of 64 records were prepared for screening on pre-formulated inclusion and exclusion criteria using the co-author-principle. During primary screening of titles and abstracts, 41 studies were excluded based on pre-established criteria, and 21 advanced to full-text review. Six of those were further excluded due to lack of pathophysiological focus, genetic-only perspectives, or methodological duplicates, leaving 15 papers for final inclusion. These 15 included studies consisted of one case–control study, one case report, one genetic case study, three in vitro/ex vivo experimental studies, one multi-level tissue analysis, one clinical observational study, and seven review papers. Details of the included studies are provided in Supplementary File 3, and the study selection process (PRISMA flow diagram) is summarized in Supplementary File 2.

### Quality assessment

Narrative quality assessment was employed to address quality and risk-of-bias considerations using criteria appropriate for mechanistic evidence in lipedema research, as lipedema research is at an early investigative stage with limited large-scale studies. This narrative approach allows for appropriate evaluation of diverse evidence types including in vitro experiments, genetic case reports, and hypothesis-generating reviews. Quality considerations are integrated throughout the Results and Discussion sections when interpreting findings, enabling context-specific assessment rather than applying standardized numerical scoring.

## Results

This systematic literature review analyzed 15 included publications. Both empirical findings and mechanistic hypotheses were treated as primary evidence and integrated through narrative synthesis to construct a comprehensive pathophysiological framework for lipedema. Although all are dedicated to the same topic, they differ in terms of the underlying pathophysiological concepts. For clarity and consistency, key constructs are operationally defined as follows: Hormonal imbalance encompasses alterations in circulating hormone levels, receptor expressions or functions, hormone metabolism (e.g., growth hormone), or signaling pathway activity. Estrogen receptor dysregulation denotes altered ERα and/or ERβ expressions or functional activity. Metabolic imbalance in the context of adipose-derived stem cells involves dysregulation of key adipogenic regulators, including PPARγ (peroxisome proliferator-activated receptor gamma), a nuclear receptor transcription factor serving as master regulator of adipocyte differentiation, and leptin, an adipokine involved in energy homeostasis and fat metabolism. Based on their hypotheses, the publications are separated into the following four pathophysiological categories and will be discussed in the corresponding sections.

### General hormonal imbalance

A number of studies suggest a distinctive hormonal profile in women with lipedema compared to healthy individuals. The concept of lipedema mimicking a pseudopregnancy hormonal state has been proposed, characterized by increased levels of estrogen, progesterone, prolactin, and relaxin [10]. This hormonal constellation promotes gluteofemoral fat accumulation while simultaneously inhibiting lipolysis—conditions that correspond with the typical phenotype observed in lipedema patients [10]. Supporting this hormonal model, it has been emphasized that lipedema is not solely a disorder of excessive fat accumulation, but rather a polygenic and hormonal contribution associated with microvascular and lymphatic dysfunctions [9]. The author highlighted hypertrophy and hyperplasia of adipocytes combined with altered angiogenic pathways, many of which may be modulated by estrogen-related genes [9]. Further evidence of a distinct hormonal and metabolic environment is shown in a case–control study, in which specific circulating parameters in women with lipedema compared to age- and BMI-matched non-lipedema women could be considered. Their study found lower levels of HbA1c as a sign for preserved glycemic control—alongside an increase in levels of adiponectin, ALAT, total, and LDL-cholesterol. Additionally, elevation of markers for inflammatory and oxidative stress could be found. However, these biomarkers lacked diagnostic specificity to distinguish lipedema from other metabolic disorders [8]. Concluded, these observations lead to the idea of lipedema being driven by a certain constellation of hormonal imbalance in the context of a multifactorial genesis, contributing to its characteristic fat distribution and resistance to conventional weight loss strategies.

### Changes in growth hormone balance

The case report of a family affected by lipedema associated with Pit1 mutation leading to GH and testosterone deficiency lays the foundation for a new association of two rare conditions [16].

This supports the idea of testosterone being a preventing factor against lipedema in men and GH deficiency leading to less lipolysis. Although lipedema is a predominantly female condition, male cases of lipedema have been reported, only connected to hepatic cirrhosis or hormonal therapy for prostatic carcinoma. These conditions are accordingly based on alterations in the male hormonal state, although they are not yet confirmed by any other author [16].

### Metabolic imbalance and adipose stem cells (ASCs)

Recent studies have highlighted the involvement of adipose-derived stem cells (ASCs) and stromal vascular fraction (SVF) in the pathophysiology of lipedema, underscoring the metabolic dysregulation within affected adipose tissue. An in vitro experimental study indicated that ASCs isolated from the thigh region (SVF-T) in lipedema patients exhibit a significantly higher adipogenic differentiation potential compared to those from the abdominal region (SVF-A) and from healthy controls [17]. This was accompanied by a proliferation of Ki67 + and CD43 + progenitor cells and an increase in hypertrophic adipocytes, indicating an upregulated adipogenic process. Furthermore, lipedema adipose tissue was characterized as highly vascularized and fibrotic with notable macrophage infiltration. At the molecular level, leptin gene expression was markedly elevated in adipocytes derived from SVF-T, while PPARγ expression was increased in SVF-A-derived adipocytes, suggesting depot-specific metabolic alterations potentially influenced by estrogen receptor signaling pathways that are known to modulate PPARγ activity [17]. In contrast, another in vitro experimental study observed a reduced response to adipogenic stimulation in lipedema-derived ASCs, alongside altered adipokine profiles and decreased aromatase expression [2]. These findings suggest an early stage impairment in differentiation and point toward a phenotype distinction between lipedema and healthy adipose tissue, though in vivo validation remains pending [2]. Complementary to this, an experimental comparative study employed gene expression arrays and cytokine quantification to examine the morphologic and molecular landscape of lipedema adipose tissue [7]. Their results revealed a unique adipose architecture distinct from obesity or lymphedema, characterized by fibrosis, adipocyte hypertrophy, and aberrant lipid metabolism. Additionally, differentially expressed genes associated with macrophages were identified, further corroborated by increased macrophage presence on histological analysis, while the T-cell compartment remained unchanged [7]. Together, these studies emphasize the distinct metabolic and inflammatory environment of lipedema tissue and suggest that dysregulated ASC behavior and adipokine signaling—potentially modulated by sex hormones—may contribute to the disease’s development and persistence.

### Changes in estrogen metabolism and receptor function

The crucial role of female sex hormones—particularly estrogen and progesterone—in the development and progression of lipedema is consistently highlighted across multiple studies. Estrogen promotes cellular proliferation, vasodilatation, and increased extracellular fluid accumulation, while progesterone contributes to tissue differentiation and enhanced energy metabolism, mimicking physiological states such as pregnancy [10]. Together, these hormones foster the typical gynoid fat distribution in the buttocks and thighs, which coincides with key hormonal transition phases, such as puberty, pregnancy, and menopause—periods frequently associated with the onset or exacerbation of lipedema symptoms [11]. A significant genetic component has been proposed by identifying a missense variant in the AKR1C1 gene (encoding an aldo–keto reductase that catalyzes progesterone inactivation) in a family with autosomal dominant non-syndromic lipedema [6]. This loss-of-function mutation was linked to impaired progesterone clearance, increased subcutaneous fat deposition, and enhanced lipogenic activity via ADD1/SREBP1c upregulation. Furthermore, AKR1C1’s role in prostaglandin synthesis implies that its dysfunction could reduce PGF2 levels, thus facilitating adipogenesis [6]. A mechanistic explanation for estrogen-driven adipose dysfunction points to an altered estrogen receptor (ER) distribution—specifically an increased ERα/ERβ ratio—as a driver of enhanced adipocyte lipid and glucose metabolism [12]. This dysregulation leads to increased PPARγ activation, glucose uptake, fatty acid storage, angiogenesis, and reduced lipolysis and mitochondrial function, ultimately promoting adipose depot expansion. The authors postulate that early dysregulation of ER signaling and local paracrine estrogen production by adipocytes are critical events in lipedema pathogenesis [12]. Similarly, the estrogen-dependent nature of lipedema has been emphasized, suggesting that the condition manifests with feminine hormonal changes and involves vascular and lymphatic anomalies, peripheral nerve inflammation, and sympathetic dysregulation [4]. They identified region-specific ER patterns in adipose tissue (e.g., decreased ERα and increased ERβ in gluteal vs. abdominal fat), as well as central hypothalamic effects on appetite regulation and metabolism through estrogen-sensitive pro-opiomelanocortin pathways. These alterations may explain both fat distribution and resistance to weight loss in affected patients [4]. These findings were corroborated by the demonstration of pathological ER distribution patterns—namely reduced ERα and increased ERβ—in white adipose tissue of lipedema patients [13]. This imbalance was associated with increased lipogenesis and decreased lipolysis in the gluteofemoral region, offering a mechanistic explanation for the absence of a typical menopausal shift from gynoid to android fat distribution in lipedema [13]. Additional insights into estrogen-related dysregulation were provided by the finding of upregulated aromatase and ZNF423 expression in perivascular adipose populations of lipedema patients, suggesting enhanced preadipocyte commitment [14]. They further pointed to endothelial barrier dysfunction via decreased VE-cadherin expression and endothelial leakiness mediated by the secretome of lipedema-derived SVF cells, proposing that adipose microenvironmental factors—rather than solely mature adipocytes—contribute to disease pathology. Cultured ASCs exhibited highest aromatase expression, underscoring the importance of freshly isolated cells in studying in vivo mechanisms [14]. The estrogen receptor axis also remains central in the work of Buso et al. [3], who align with Szél et al.’s [4] findings and approve ER dysregulation as a core pathomechanism in lipedema [3]. Complementing this, it has been proposed that lipedema is an estrogen-dependent adipose disorder triggered by CAV1 dysfunction [15]. They indicated that impaired CAV1 phosphorylation uncouples ERα and MMP14, increasing perivascular matrix remodeling and leading to hypertrophic subcutaneous white adipose tissue (sWAT). Additionally, disruption of the PROX1–CAV1 axis may impair lymphatic vessel integrity, contributing to the lymphovascular symptoms seen in lipedema. The cumulative effect of these pathways—enhanced ERα-sensitivity, adipocyte hypertrophy, and vascular dysfunction—further supports the hormone-dependent etiology of the disease [15].

**Fig. 1:**
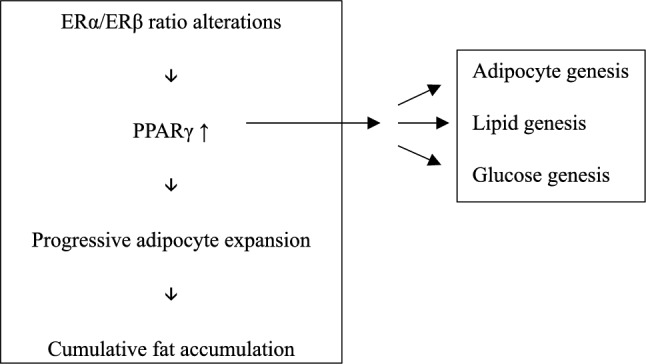
Schematic illustration of processes in adipose-derived stem cells of lipedema patients (Author’s own illustration)

The complex molecular interactions between estrogen receptor subtypes, PPARγ activation, and its influence on adipose stem cell function are summarized schematically in Fig. 1.

## Discussion

This systematic literature review yielded 15 publications to be included for highlighting the central role of hormonal influence in the development of lipedema. The analysis led to four interlinked hypotheses underlying lipedema development: systemic hormonal imbalance, altered GH and testosterone activity, metabolic dysfunction, and aberrant estrogen and estrogen receptor metabolism. A distinct hormonal imbalance, including elevated estrogen, progesterone, prolactin, and relaxin levels, appears to contribute to the characteristic fat distribution and resistance to lipolysis observed in affected women. Although circulating biomarkers differ between lipedema patients and controls, no single parameter has yet shown diagnostic specificity. Growth hormone deficiency combined with testosterone alterations was identified in rare male cases, suggesting that mentioned hormonal pathways may also modulate disease expression. In addition, metabolic dysregulation at the adipose stem cell level, characterized by altered adipogenic potential, fibrosis, and inflammatory infiltration, supports a role for local tissue dysfunction in lipedema pathophysiology. Most prominently, estrogen receptor dysregulation—particularly alterations in the distribution of ERα and ERβ—was repeatedly identified as a driving mechanism. This hormonal imbalance promotes adipocyte hypertrophy, impaired lipolysis, and vascular dysfunction, distinct from changes seen in obesity or lymphedema. Although targeted hormone modulation and ASC-based diagnostic need additional exploration [6, 15]. Alterations in the function of structural protein, such as CAV1 dysfunction, and genetic factors, such as mutations in AKR1C1, further point toward a hormone-sensitive and possibly inherited component in lipedema. In addition, Child et al. [5] are proposing lipedema to be a genetic condition with either x-linked dominant inheritance or more likely, autosomal dominant inheritance with sex limitations, corresponding to observations of an above discussed family case report [6]. The findings compiled thus lay the foundation for further research in the field of lipedema.

Evidence certainty across the four hypotheses varies considerably. Strong evidence supports estrogen receptor dysregulation through repeated reporting in multiple studies. Moderate evidence seems to exist for hormonal profile alterations, and PPARγ/leptin dysregulations, with experimental support requiring independent validation in larger cohorts. Evidence for growth hormone as well as for genetic factors appear to be limited, being derived from single-family case reports. The observational nature of most studies excludes strong causal conclusions, emphasizing the need for prospective research to establish definitive pathophysiological mechanisms.

Included studies are predominantly observational or preclinical, with small sample sizes, variable methodologies, and lack of replication. Further, most are not longitudinal or interventional, and few have validated their findings in vivo, limiting determination of causality direction between hormonal alterations and lipedema development. Formal risk-of-bias assessment tools were not applicable given heterogeneous study designs; narrative quality assessment lacks standardization and sensitivity analyses were not performed. The differential diagnosis challenge is a critical limitation when interpreting hormonal findings. Clinically, lipedema frequently co-occurs with obesity, yet no hormonal or molecular markers have been identified that reliably distinguish lipedema from obesity in comparative studies. This overlap makes it impossible to determine whether observed hormonal patterns are lipedema-specific or simply reflect obesity-related confounding. Differentiation from lymphedema appears clearer from a hormonal perspective, as lymphedema results from mechanical lymphatic disruption. However, the clinical relationship remains complex, whether lipedema and lymphedema coexist as independent conditions or whether lipedema's pathophysiology secondarily causes lymphatic compromise. Clinical features (e.g., feet-sparing) have not been systematically correlated with molecular findings. Cross-study confounding factors further complicate interpretation. Menopausal status, contraceptive use, and reproductive history, key determinants of hormonal profiles, were inconsistently reported, preventing adequate control for these confounders. Searches were conducted April 5, 2023. Therefore, newer evidence in this rapidly evolving field may not be captured. Although the number of included publications was limited, this circumstance enabled to maintain a clear overview of all included publications throughout the whole search process while preventing overlooking any information of potential relevance. This limited number of analyzed publications further allowed to simplify the data extraction process and to detect the interference between some of the publications as authors were referring to each other’s works.

## Conclusion

This systematic literature review highlights the multifaceted nature of lipedema, pointing to a complex interplay between hormonal, metabolic, and potentially genetic factors in its pathogenesis. Estrogen and progesterone, along with their receptor-mediated pathways, appear to play a central role in driving adipocyte dysfunction, impaired lipolysis, and abnormal fat distribution—particularly through altered ERα/ERβ ratios that favor adipose expansion. Additionally, these results suggest that disruptions in adipose stem cell behavior, growth hormone balance, and metabolic signaling pathways involving leptin and PPARγ may contribute to the disease’s onset and progression. Although current findings remain largely preclinical and diverse, the consistency in identifying estrogen-related dysregulation across studies underscores the potential for developing hormone-based diagnostic tools and targeted therapeutics. Future investigations should prioritize the validation of multi-parametric biomarker panels combining circulating hormones, metabolic markers, and genetic screening (AKR1C1, CAV1 variants) to improve diagnostic accuracy and enable earlier detection. Such validated biomarkers are particularly critical for differential diagnosis, as current hormonal markers cannot reliably distinguish lipedema from obesity in clinical practice. Furthermore, future clinical research should focus on large-scale, controlled studies evaluating sex hormone profiles—especially across life transitions, such as puberty, pregnancy, and menopause. To ensure that further insights extend from diagnostics to therapy, the development of estrogen receptor-targeted interventions should be evaluated through randomized controlled trials. However, translating these mechanistic insights into clinical practice fundamentally depends on a paradigm shift in how lipedema is conceptualized. Recognizing lipedema as a hormonally sensitive disorder distinct from obesity is essential to improving diagnostic accuracy, reducing stigma, and guiding more effective interventions.

## Supplementary Information

Below is the link to the electronic supplementary material.

Supplementary file1 (PDF 107 KB)

Supplementary file2 (PDF 63 KB)

Supplementary file3 (XLSX 32 KB)

## Data Availability

No datasets were generated or analyzed during the current study.
