# Supplementary material for: Impact of hormones on lipedema development: a systematic literature review
Source: Arch Gynecol Obstet. 2026 Jan 23;313(1):60. doi: 10.1007/s00404-026-08318-1 (PMC12830482; doi:10.1007/s00404-026-08318-1)
Supplement: Supplementary file 1 — Supplementary file1 (PDF 107 KB) [file 404_2026_8318_MOESM1_ESM.pdf]

Hormones and Lipedema

Date of last run search: April 5th 2023

Petra Stute, Julia Lüchinger, Cynthia Giachino

Information Sources and Content Coverage Dates

- MEDLINE (R) ALL (Ovid), Coverage 1946-Present
- Embase (Ovid), Coverage 1947-Present
- Cochrane Library (Wiley), Coverage, 1996-Present
- Science Citation Index Expanded (SCIE) & Emerging Sources Citation Index (ESCI), (Web of Science), Coverages, 1900-Present & 2017-Present

Search strategy & deduplication process

The literature search was performed by combining controlled vocabulary terms (e.g., MeSH, EMTREE,) and free text terms for the following concepts: hormones and lipedema. No language or publication date limits were applied. Search strategies were translated accordingly for each information source. A total of 121 records were retrieved. Duplicates were removed with Deduklick, a fully automated deduplication software. A new total of 64 records were prepared in Covidence for screening.

Reference: Borissov N, Haas Q, Minder B, Kopp-Heim D, von Gernler M, Janka H, Teodoro D, Amini P. Reducing systematic review burden using Deduklick: a novel, automated, reliable, and explainable deduplication algorithm to foster medical research. Systematic reviews. 2022 Dec;11(1):1-0. https://pubmed.ncbi.nlm.nih.gov/35978441/

MEDLINE (R) ALL (Ovid)

Ovid MEDLINE(R) ALL <1946 to April 04, 2023>

1 exp Hormones/ or (hormon* or "Adrenal Cortex Hormone?" or "Anti-Mullerian hormone?" or "17-Ketosteroids" or "17 oxosteroids" or Androstenedione or Androsterone or epiandrosterone or Dehydroepiandrosterone or dehydroisoandrosterone or androstenolone or dhea or prasterone or "Follicle-stimulating hormone?" or follitropin? or FSH or Estrone or estrovarin or kestrone or Etiocholanolone or Glucocorticoid? or Hydroxycorticosteroid? or "11-Hydroxycorticosteroid?" or "17-Hydroxycorticosteroid?" or Desoxycorticosterone or deoxycorticosterone or desoxycortone or Pregnenolone or "Gastrointestinal Hormone?" or "enteric hormone?" or "intestinal hormone?" or Cholecystokinin or pancreozymin or uropancreozymin or Sincalide or syncalide or "Epidermal Growth Factor?" or "beta urogastrone" or "Gastric Inhibitory Polypeptide?" or "Gastrin-Releasing Peptide?" or Gastrins or Pentagastrin or peptavlon or "gastrin pentapeptide?" or Tetragastrin or Motilin or "Peptide YY" or Proglucagon or Secretin? or "Vasoactive Intestinal Peptide?" or "Gonadal Hormone?" or Activins or "Corpus Luteum Hormone?" or "Gonadal Steroid Hormone?" or Inhibins or "Testicular Hormone?" or "Ectopic hormone?" or Melatonin or "Peptide Hormone?" or Adiponectin or Leptin? or Resistin or "Zn-Alpha-2-Glycoprotein Activins" or "Inhibin-beta Subunits" or Adipokines or Adrenomedullin or Angiotensins or Bombesin or Calcitonin or Ghrelin or Gonadotropin? or "Hypothalamic Hormone?" or Inhibins or Motilin or "Natriuretic Peptide?" or "Pancreatic Hormone?" or progestogen? or progestin? or progestagen? or Estrogen? or "Parathyroid Hormone?" or "Peptide PHI" or "Pituitary Hormone?" or "Placental Hormone?" or Procalcitonin or Progranulins or Relaxin or Urocortins or Urotensins or "Vasoactive Intestinal Peptide?" or "Thymus Hormone?" or "Circulating Thymic Factor?" or Thymopoietin? or Thymosin? or "Thyroid Hormone?" or Calcitonin or thyrocalcitonin or calcitrin or Dextrothyroxine or Diiodotyrosine or Monoiodotyrosine or Thyronine? or Thyroxin?).ti,ab,kf,nm. 1988433

2 Lipedema/ or (lip?edema* or lipolymph?edema* or lipo-lymph?edema*).ti,ab,kf. 465

3 1 and 2 35

4 ("36479502" or "35625899" or "35448699" or "34660173" or "36401222" or "36387874" or "36142221" or "34769153").ui. 8

5 3 and 4 8

[Note: Line 4 are key papers, line 5 shows key papers are retrieved by the search]

Embase (Ovid)

Embase <1974 to 2023 April 04>

1 exp hormone/ or (hormon* or "Adrenal Cortex Hormone?" or "Anti-Mullerian hormone?" or "17-Ketosteroids" or "17 oxosteroids" or Androstenedione or Androsterone or epiandrosterone or Dehydroepiandrosterone or dehydroisoandrosterone or androstenolone or dhea or prasterone or "Follicle-stimulating hormone?" or follitropin? or FSH or Estrone or estrovarin or kestrone or Etiocholanolone or Glucocorticoid? or Hydroxycorticosteroid? or "11-Hydroxycorticosteroid?" or "17-Hydroxycorticosteroid?" or Desoxycorticosterone or deoxycorticosterone or desoxycortone or Pregnenolone or "Gastrointestinal Hormone?" or "enteric hormone?" or "intestinal hormone?" or Cholecystokinin or pancreozymin or uropancreozymin or Sincalide or syncalide or "Epidermal Growth Factor?" or "beta urogastrone" or "Gastric Inhibitory Polypeptide?" or "Gastrin-Releasing Peptide?" or Gastrins or Pentagastrin or peptavlon or "gastrin pentapeptide?" or Tetragastrin or Motilin or "Peptide YY" or Proglucagon or Secretin? or "Vasoactive Intestinal Peptide?" or "Gonadal Hormone?" or Activins or "Corpus Luteum Hormone?" or "Gonadal Steroid Hormone?" or Inhibins or "Testicular Hormone?" or "Ectopic hormone?" or Melatonin or "Peptide Hormone?" or Adiponectin or Leptin? or Resistin or "Zn-Alpha-2-Glycoprotein Activins" or "Inhibin-beta Subunits" or Adipokines or Adrenomedullin or Angiotensins or Bombesin or Calcitonin or Ghrelin or Gonadotropin? or "Hypothalamic Hormone?" or Inhibins or Motilin or "Natriuretic Peptide?" or "Pancreatic Hormone?" or progestogen? or progestin? or progestagen? or Estrogen? or "Parathyroid Hormone?" or "Peptide PHI" or "Pituitary Hormone?" or "Placental Hormone?" or Procalcitonin or Progranulins or Relaxin

or Urocortins or Urotensins or "Vasoactive Intestinal Peptide?" or "Thymus Hormone?" or "Circulating Thymic Factor?" or Thymopoietin? or Thymosin? or "Thyroid Hormone?" or Calcitonin or thyrocalcitonin or calcitrin or Dextrothyroxine or Diiodotyrosine or Monoiodotyrosine or Thyronine? or Thyroxin?).ti,ab,kf. 1424672

2 lipedema/ or (lip?edema* or lipolymph?edema* or lipo-lymph?edema*).ti,ab,kf. 860

3 1 and 2 50

Cochrane Library (Wiley)

Advanced Search Mode

#1 [mh hormones] or (hormon* or (Adrenal NEXT Cortex NEXT Hormone?) or (Anti-Mullerian NEXT hormone?) or "17-Ketosteroids" or "17 oxosteroids" or Androstenedione or Androsterone or epiandrosterone or Dehydroepiandrosterone or dehydroisoandrosterone or androstenolone or dhea or prasterone or (Follicle-stimulating NEXT hormone?) or follitropin? or FSH or Estrone or estrovarin or kestrone or Etiocholanolone or Glucocorticoid? or Hydroxycorticosteroid? or "11-Hydroxycorticosteroid" or "11-Hydroxycorticosteroids" or "17-Hydroxycorticosteroid" or "17-Hydroxycorticosteroids" or Desoxycorticosterone or deoxycorticosterone or desoxycortone or Pregnenolone or (Gastrointestinal NEXT Hormone?) or (enteric NEXT hormone?) or (intestinal NEXT hormone?) or Cholecystokinin or pancreozymin or uropancreozymin or Sincalide or syncalide or (Epidermal NEXT Growth NEXT Factor?) or "beta urogastrone" or (Gastric NEXT Inhibitory NEXT Polypeptide?) or (Gastrin NEXT Releasing NEXT Peptide?) or Gastrins or Pentagastrin or peptavlon or (gastrin NEXT pentapeptide?) or Tetragastrin or Motilin or "Peptide YY" or Proglucagon or Secretin? or (Vasoactive NEXT Intestinal NEXT Peptide?) or (Gonadal NEXT Hormone?) or Activins or (Corpus NEXT Luteum NEXT Hormone?) or (Gonadal NEXT Steroid NEXT Hormone?) or Inhibins or (Testicular NEXT Hormone?) or (Ectopic NEXT hormone?) or Melatonin or (Peptide NEXT Hormone?) or Adiponectin or Leptin? or Resistin or "Zn-Alpha-2-Glycoprotein Activins" or "Inhibin-beta Subunits" or Adipokines or Adrenomedullin or Angiotensins or Bombesin or Calcitonin or Ghrelin or Gonadotropin? or (Hypothalamic NEXT Hormone?) or Inhibins or Motilin or (Natriuretic NEXT Peptide?) or (Pancreatic NEXT Hormone?) or progestogen? or progestin? or progestagen? or Estrogen? or (Parathyroid NEXT Hormone?) or "Peptide PHI" or (Pituitary NEXT Hormone?) or (Placental NEXT Hormone?) or Procalcitonin or Progranulins or Relaxin or Urocortins or Urotensins or (Vasoactive NEXT Intestinal NEXT Peptide?) or (Thymus NEXT Hormone?) or (Circulating NEXT Thymic NEXT Factor?) or Thymopoietin? or Thymosin? or (Thyroid NEXT Hormone?) or Calcitonin or thyrocalcitonin or calcitrin or Dextrothyroxine or Diiodotyrosine or Monoiodotyrosine or Thyronine? or Thyroxin?):ti,ab,kw 136392

#2 [mh lipedema] or (lip?edema* or lipolymph?edema* or lipo-lymph?edema*):ti,ab,kw 28

#3 #1 AND #2 4

Science Citation Index Expanded (SCIE) & Emerging Sources Citation Index (ESCI), (Web of Science)

#	Search Query	Database	Results
1	TS=(hormon* OR "Adrenal Cortex Hormone$" OR "Anti-Mullerian hormone$" OR "17-Ketosteroids" OR "17 oxosteroids" OR Androstenedione OR Androsterone OR epiandrosterone OR Dehydroepiandrosterone OR dehydroisoandrosterone OR androstenolone OR dhea OR prasterone OR "Follicle-stimulating hormone$" OR follitropin$ OR FSH OR Estrone OR estrovarin OR kestrone OR Etiocholanolone OR Glucocorticoid$ OR Hydroxycorticosteroid$ OR "11-Hydroxycorticosteroid$" OR "17-Hydroxycorticosteroid$" OR Desoxycorticosterone OR deoxycorticosterone OR desoxycortone OR Pregnenolone OR "Gastrointestinal Hormone$" OR "enteric hormone$" OR "intestinal hormone$" OR Cholecystokinin OR pancreozymin OR uropancreozymin OR Sincalide OR syncalide OR "Epidermal Growth Factor$" OR "beta urogastrone" OR "Gastric Inhibitory Polypeptide$" OR "Gastrin-Releasing Peptide$" OR Gastrins OR Pentagastrin OR peptavlon OR "gastrin pentapeptide$" OR Tetragastrin OR Motilin OR "Peptide YY" OR Proglucagon OR Secretin$ OR "Vasoactive Intestinal Peptide$" OR "Gonadal Hormone$" OR Activins OR "Corpus Luteum Hormone$" OR "Gonadal Steroid Hormone$" OR Inhibins OR "Testicular Hormone$" OR "Ectopic hormone$" OR Melatonin OR "Peptide Hormone$" OR Adiponectin OR Leptin$ OR Resistin OR "Zn-Alpha-2-Glycoprotein Activins" OR "Inhibin-beta Subunits" OR Adipokines OR Adrenomedullin OR Angiotensins OR Bombesin OR Calcitonin OR Ghrelin OR Gonadotropin$ OR "Hypothalamic Hormone$" OR Inhibins OR Motilin OR "Natriuretic Peptide$" OR "Pancreatic Hormone$" OR progestogen$ OR progestin$ OR progestagen$ OR Estrogen$ OR "Parathyroid Hormone$" OR "Peptide PHI" OR "Pituitary Hormone$" OR "Placental Hormone$" OR Procalcitonin OR Progranulins OR Relaxin OR Urocortins OR Urotensins OR "Vasoactive Intestinal Peptide$" OR "Thymus Hormone$" OR "Circulating Thymic Factor$" OR Thymopoietin$ OR Thymosin$ OR "Thyroid Hormone$" OR Calcitonin OR thyrocalcitonin OR calcitrin OR Dextrothyroxine OR Diiodotyrosine OR Monoiodotyrosine OR Thyronine$ OR Thyroxin$) Editions: WOS.SCI,WOS.ESCI	Web of Science Core Collection	1341267
2	TS=(lip$edema* OR lipolymph$edema* OR lipo-lymph$edema*) Editions: WOS.SCI,WOS.ESCI	Web of Science Core Collection	480
3	#2 AND #1 Editions: WOS.SCI,WOS.ESCI	Web of Science Core Collection	32
