# Supplementary material for: Impact of hormones on lipedema development: a systematic literature review
Source: Arch Gynecol Obstet. 2026 Jan 23;313(1):60. doi: 10.1007/s00404-026-08318-1 (PMC12830482; doi:10.1007/s00404-026-08318-1)
Supplement: Supplementary file 2 — Supplementary file2 (PDF 63 KB) [file 404_2026_8318_MOESM2_ESM.pdf]

PRISMA 2020 flow diagram for new systematic reviews which included searches of databases and registers only

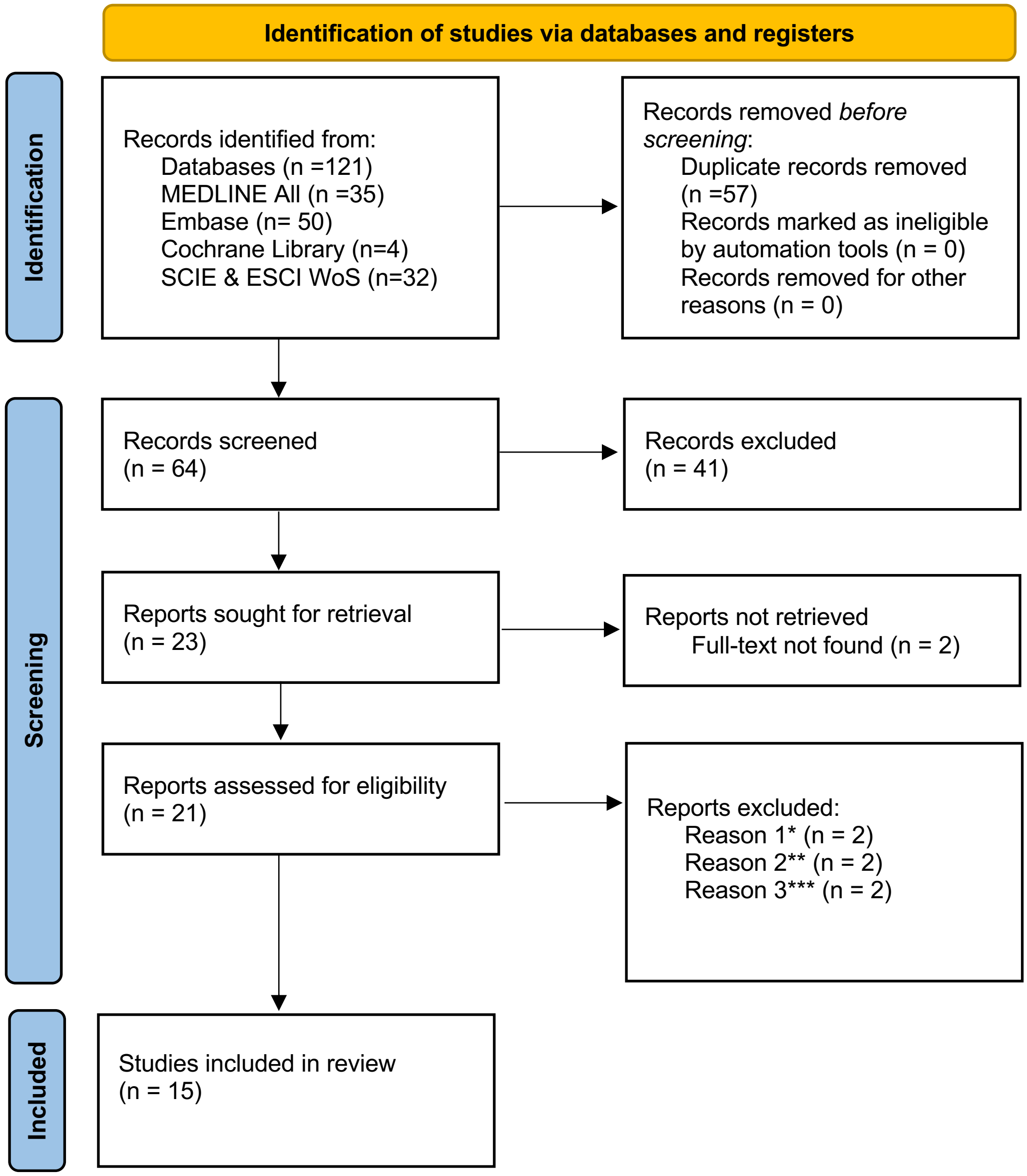

comments:
** missing pathophysiological theory*
*** same paper as another*
**** wrong outcome*

*Consider, if feasible to do so, reporting the number of records identified from each database or register searched (rather than the total number across all databases/registers).

**If automation tools were used, indicate how many records were excluded by a human and how many were excluded by automation tools.

From: Page MJ, McKenzie JE, Bossuyt PM, Boutron I, Hoffmann TC, Mulrow CD, et al. The PRISMA 2020 statement: an updated guideline for reporting systematic reviews. BMJ 2021;372:n71. doi: 10.1136/bmj.n71

For more information, visit: http://www.prisma-statement.org/
